# “Just Carbon”: Ideas About Graphene Risks by Graphene Researchers and Innovation Advisors

**DOI:** 10.1007/s11569-018-0324-y

**Published:** 2018-10-22

**Authors:** Rickard Arvidsson, Max Boholm, Mikael Johansson, Monica Lindh de Montoya

**Affiliations:** 1 Division of Environmental Systems Analysis, Chalmers University of Technology, Vera Sandbergs Allé 8, 412 96 Gothenburg, Sweden; 2 Gothenburg Research Institute, University of Gothenburg, PO Box 603, 405 30 Gothenburg, Sweden

**Keywords:** Responsible research and innovation, ELSA, Nanomaterial, Safety, Risk association

## Abstract

Graphene is a nanomaterial with many promising and innovative applications, yet early studies indicate that graphene may pose risks to humans and the environment. According to ideas of responsible research and innovation, all relevant actors should strive to reduce risks related to technological innovations. Through semi-structured interviews, we investigated the idea of graphene as a risk (or not) held by two types of key actors: graphene researchers and innovation advisors at universities, where the latter are facilitating the movement of graphene from the laboratory to the marketplace. The most common idea found is that graphene is not a risk due to, e.g., low toxicity, low amounts produced/used, and its similarity to harmless materials (being “just carbon”). However, some researchers and advisors also say that graphene is a risk, e.g., under certain conditions or due to a lack of risk-related information. We explain the co-existence of these seemingly contradictory ideas through (1) the semantic ambiguity of the word risk and (2) a risk/no-risk rhetoric, where risks are mentioned rhetorically only to be disregarded as manageable or negligible. We suggest that some of the ideas held by the researchers and innovation advisors constitute a challenge to responsible research and innovation regarding graphene. At the same time, we acknowledge the dilemma that the discourse of responsible innovation creates for the actors: denying graphene risks makes them irresponsible due to a lack of risk awareness, while affirming graphene risks makes them irresponsible due to their everyday engagement in graphene development. We therefore recommend more research into what researchers and innovation advisors should do in practice in order to qualify as responsible.

## Introduction

Graphene has risen as a new star on the technological sky. In 2010, Andre Geim and Konstantin Novoselov were awarded the Nobel Prize for their “groundbreaking experiments” regarding the carbon-based nanomaterial graphene [1]. Graphene has a number of extraordinary properties, including high electric mobility [2], high thermal conductivity [3], high strength [4] and antibacterial properties [5]. Because of these properties, it is alleged to have a wide range of innovative applications, including improved electronics [6], in particular thin, light, and bendable displays [7]; improved energy storage, such as highly effective batteries [8]; material enhancement, including stronger as well as electronically and thermally conductive polymer materials [9]; and medical utilities, such as new antibacterial agents [10]. Whereas these applications are extraordinary enough, there are also expectations of even more revolutionary innovations not yet conceptualized but waiting for discovery in the future. Thus, graphene is often referred to as a “wonder material,” “miracle material,” and “supermaterial” in scientific papers and the public discourse [11, 12]. To fully harness the promises of graphene, several research programs have emerged around the world with the purpose of guaranteeing the successful realization of its potential. For example, the European Union (EU) finances a research program called the Graphene Flagship, with a total budget of 1 billion Euros. This is one of the EU’s largest research initiatives ever. On the program’s website, it says [13]:

“The Graphene Flagship fosters the emergence of foundational breakthroughs in graphene science and technologies and develops new engineering concepts to exploit the unique opportunities offered by graphene and its derivatives.”

The Flagship, launched in 2013, involves more than 150 academic and industrial research groups in 23 countries and is coordinated by Chalmers University of Technology in Gothenburg, Sweden. Given the impact that graphene research “is expected to exert on tomorrow’s technologies and world economy,” among the objectives of the Graphene Flagship are “to build a pathway for the newly accumulated strategic knowledge to impact European industries and society” and “to secure a major role [of the EU] in this ongoing technological revolution” [13].

As the above paragraph makes clear, the hopes for graphene are high. Yet, while technological innovations may bring huge benefits, they may also cause risk to contemporary societies [14, 15]. Therefore, concepts that attempt to incorporate awareness and mitigation of the unwanted side effects of technological innovation have been developed. One such concept is responsible innovation [16], often extended to responsible research and innovation (RRI) [17]. RRI builds on a number of already existing concepts and perspectives, such as ethical, legal, and societal implications or aspects (ELSI/A); technology assessment; applied ethics; as well as science and technology studies [18, 19]. An important feature of RRI is that scientists should be morally responsible for the societal impacts and risks that emerging technologies may bring [20]. Several definition of RRI have been proposed [21], for example, one by von Schomberg [22]:

“Responsible Research and Innovation is a transparent, interactive process by which societal actors and innovators become mutually responsive to each other with a view on the (ethical) acceptability, sustainability and societal desirability of the innovation process and its marketable products (in order to allow a proper embedding of scientific and technological advances in our society).”

RRI has recently become particularly visible in policies originating from the EU and, above all, the European Commission, yet the translation of the concept into practice is ambiguous [20]. Although it seems to be challenging to define and operationalize RRI, it clearly implies that relevant actors should assume responsibility for the ethical, safe, and sustainable development of emerging technologies, in particular by steering away from severe risks.

Regarding graphene innovations specifically, risk-related research is evidently lagging behind that of graphene development and applications [23]. Although the risks of nanomaterials are surrounded by high uncertainty due to unknown exposure and toxicity [24–26], a number of issues have already emerged for graphene. The above-mentioned Graphene Flagship has a work package dedicated to the health- and environmentally related issues of graphene, acknowledging that “the small size and unique physio-chemical properties of graphene pose potential risks to the health of animals, humans and the environment” [27]. However, no definitive results or answers regarding graphene risks seem to have emerged from the work package yet. Based on a literature review, Arvidsson et al. [28] wrote that graphene is a chemically persistent substance that could exert considerable toxicity. Another review of toxicity results showed that some studies did not indicate particular hazards related to graphene, whereas others did [29]. An especially notable study found that micrometer-thick sheets of a few graphene layers can penetrate cell membranes at the sheets’ corners and asperities, of which numerous exist along the irregular edges of fabricated graphene [30]. This piercing of cells illustrates the inherent potential of graphene to cause harm to organisms. To minimize the risks of graphene, Bussy et al. [31] provided three recommendations: (1) use small, individual graphene sheets that the body can easily dispose of, (2) use stable dispersions of graphene to minimize agglomeration of graphene sheets inside the body, and (3) use graphene materials that can be excreted or degraded effectively inside the body. Park et al. [11] provided further recommendations for how different actors (e.g., innovators, scientific experts, and risk assessors) could contribute to the safe development of graphene. Taking a broader life cycle perspective, a number of studies have noticed that the energy use in producing graphene can be high, exceeding 1000 MJ/kg for some production routes [32–34]. To compare, the relatively energy-intensive conventional material aluminum typically requires about 200 MJ/kg [35].

Studies thus point both at the potential of graphene itself to cause biological harm as well as its potential to harm society and the environment more indirectly through high energy demand. Considering these early indications of risks, this paper sets out to explore the actual implementation of ethical and responsible perspectives in the innovation process of graphene. It focuses on two actors at the very center of graphene innovation: graphene researchers and innovation advisors at universities. The aim of this paper is to describe their ideas about the potential risks of graphene. The concequences of these ideas for RRI are then discussed. A number of previous studies have investigated whether nanoscientists believe that nanomaterials require regulation, and if so, what type of regulation [36–39]. We here ask the more fundamental question of whether the actors consider graphene to be a risk, which is a similar research question to that of Bertoldo et al. [40], Johansson and Boholm [41], and Powell [42] who, however, studied scientists’ views of the risks of nanomaterials more generally.

## Method

### Data Acquisition

This study is based on two sets of data. First, interviews were conducted between April 2015 and May 2016 among scientists working with graphene at Chalmers University of Technology in Gothenburg, Sweden. As noted, this university is the coordinator of the Graphene Flagship, and also houses several other major graphene-related research projects and infrastructures, including a Graphene Center and a national innovation program for graphene, called SIO Grafen. Not all graphene researchers interviewed are part of the Graphene Flagship itself but work with graphene at the Department of Physics and the Department of Microtechnology and Nanoscience. Most are involved in one or more of the Graphene Flagship, the Graphene Center and SIO Grafen initiatives. Interviews were conducted in a semi-structured manner [43] with open-ended questions on which the interviewees could elaborate freely. The question relevant for this paper was “Do you see any risks with graphene or in the manufacturing process?” Other questions were also asked during interviews but are not considered here. All in all, 15 people were interviewed, 12 males and 3 females, which reflects the current male bias among researchers active in the field of nanoscience [36–39, 44].

Second, semi-structured interviews were carried out with six innovation advisors (five men, one woman) working on projects involving graphene. Four of these were associated with the graphene innovation programs noted above, and two were advisors at other universities in Sweden and had worked with projects involving graphene. These innovation advisors are tasked with facilitating the movement of graphene from the laboratory to the marketplace. They are trained scientists themselves, with doctorates in a variety of scientific and engineering fields, and most often with commercial experience as well. They are the starting point of a number of intermediaries between the scientists and marketable applications that will reach a wider public. While these interviews concerned the innovation process and the commercialization of nanotechnology in general, questions were included that focused on graphene, including the possible risks that it might entail, for example: “Do you see any risks [with graphene]?”

### Data Categorization

The interviews of the graphene researchers and innovation advisors were transcribed and the material coded into categories in an iterative process. The primary focus was the categorization of the respondents’ main ideas about graphene risks, i.e., the respondents’ risk associations with graphene. By risk association, an actor establishes a connection between something and the notion of risk [45–47]. In our case, that something is graphene and we are interested in respondents’ representation of graphene as either a risk, or not a risk.

The notion of risk is multifaceted and has received theoretical interest from a variety of disciplines [48]. Many definitions establish two essential elements: evaluation and uncertainty [49–52]. Risk is an evaluative concept since statements of risk presuppose that some value is at stake. As such, the concept of risk has a subjective component: it presupposes some agent (a subject) from whose point of view something of value is jeopardized. Risk also presupposes uncertainty in the sense that some aspect of the future is not perfectly known.

In supposed contrast to this subjective understanding of risk, claims are sometimes made that risk is objective [49, 53, 54]. There are at least two interpretations of this notion. First, the term risk in common usage also refers to an unwanted event (e.g., developing cancer, injuries, and fatalities) and the cause of an unwanted event (e.g., chemicals and human behavior) [50], which are both real. Second, risk is often defined in quantitative terms, for example, as the probability of an unwanted event or as the mathematical product of probability and consequence of an unwanted event [55–57]. Given frequentist conceptions of probability, risk then becomes an objective measure of occurrences in the past (e.g., hours of exposure).

In response to such ideas of objective risk, we note that the frequentist understanding of probability has been criticized for various reasons [58]. We also argue that even though cancer and chemicals are real, referring to them as risks nevertheless involves a subjective component. We therefore assume in this paper that risk associations essentially are subjective in the sense that statements of the type “*x* is a risk” presuppose a perspective from which some value is at stake. Still, such statements may be motivated by objective reasons, including evidence that human exposure to *x* can cause cancer [49].

In addition to associations between graphene and risk, we are interested in the arguments that support such risk claims and we therefore identify the rationales communicated [59]. Some responses by the respondents were found to follow a risk/no-risk rhetoric as described by Corvellec and Boholm [60] and therefore categorized accordingly. Corvellec and Boholm [60] noted that risks described in environmental impact assessment reports of offshore wind power farms were often first associated with risk, but later on disconnected and dissociated from risk, claiming risks to be nonexistent, negligible, or manageable.

## Results

**Fig. 1:**
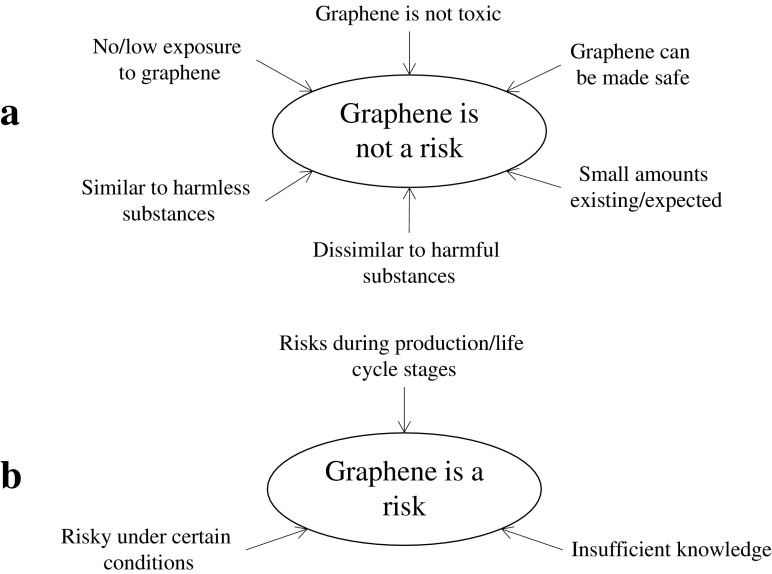
**a**, **b** Summary of the interviewed graphene researchers’ and innovation advisors’ two most widely voiced primary ideas about graphene risks (encircled) and the rationales provided (at the start of the arrows)

Concerning risk associations of graphene, there are two main ideas communicated by the graphene researchers and innovation advisors interviewed: (1) graphene is not a risk and (2) graphene is a risk. Sometimes, these two seemingly contradictory views are communicated by the same respondent, although not necessarily in an inconscient way. These views are described in more detail below, along with the rationales provided by the respondents. Figure 1 provides a summary of the most widely voiced primary ideas and their rationales. It can be noted that these rationales correlate well with arguments identified in a previous study of antibacterial silver as a risk issue, including high/low toxicity, high/low exposure, low amount/quantity, and similarity to harmful substances (in that case mercury) [59].

### Graphene Is Not a Risk

There were many general assertions by the graphene researchers saying or implying that graphene is not a risk, neither to humans nor to the environment, such as “it [graphene] seems to be safe,” “there are no risks with graphene,” “I trust my colleagues who say that there is no risk with graphene,” “it is a safe material,” “graphene is not really a problem in nature,” “we see nothing alarming,” “the consumer has minimal risk,” and “graphene […] is environmentally friendly”. One researcher said:

“It is not clear how toxic graphene can be. If you change graphene with molecules it will be safe. If you insert graphene into your body it might hurt you. But you don’t insert it into the body.”

This quote suggests that the absence of risk is easily achieved, e.g., by chemical modifications or simply by not (consciously) inserting it into the human body. More specific statements provide graphene’s lack of toxicity as a rationale for its lack of risk: “graphene will not attack cells” and “it is not toxic”. Other claims provide the lack of exposure to graphene as rationale, referring to the presence of safety equipment in labs (masks and fume hoods), the fast excretion of graphene from the body, and graphene being encapsulated in products. Another rationale stated for a lack of risk is the small amounts of manufactured graphene currently existing and anticipated in the future. One researcher said:

“If we replaced all electronic screens in the world, 2 billion touchscreens per year, there would be 60 kilograms emissions of graphene. This is not much.”

Another frequent rationale for graphene not being risky is its similarity to other harmless substances, in particular the graphite carbon in lead pencils. Quotes include “carbon is not dangerous,” “I have no worries touching it, it is like pencils, graphite,” “it does not pollute as it is just carbon,” “regarding risks, graphene should not be that dangerous, it is graphite,” and “graphene is carbon; it is not dangerous for nature”. One researcher said:

“Since the 1850s all schoolchildren have used [lead] pencils, they have ingested graphene. Since we learned to make fire we have ingested graphene from the ashes.”

The basis for this rationale is that the carbon form graphite found in lead pencils and ashes consist of graphene sheets, although separate graphene sheets are considered to be another carbon form (allotrope) than graphite.

A similar rationale is that graphene is not a risk because of its similarity to the presumed harmless material silicon, as exemplified by this quote:

“To manufacture graphene you use Scotch tape, it is the same procedure to produce silicon. There is no danger. Not worse than silicon.”

The ideas about graphene risk voiced by innovation advisors echoed those of the scientists in many respects. All pointed out that graphene likely poses no risk, in particular due to lack of toxicity: “at the moment it seems that there’s no risk, at least this far we haven’t found any specific risk,” “if it’s handled in a proper way, nothing too worrying has come out,” “one can say that it’s not poisonous, there’s no danger that it is a toxin,” and “no risks that I know of,” “at this time it’s not problematic because we haven’t found anything yet.” One advisor elaborated:

“So far we haven’t seen any larger risks, on the contrary, those who are researching the risks […] think that their work is very boring, as they can only publish when they find risks. The toxicologists look for problems and when they can’t find any they can’t publish, so it’s too bad for them – but it’s good, of course that they don’t find anything. So far there are no studies that indicate danger.”

This lack of discovered toxicity in graphene by toxicologists was also pointed out by a second advisor. Yet, another advisor said that while there were some doubts about the long-term effects of its use in the human body or with other living creatures, he saw no risks in industrial applications, and that risks had not been discussed—they, the advisors, depended on the common sense of the researchers they worked with, and on the reviews that the research had undergone.

The claim that graphene is not a risk if proper procedures are followed came up. For example, one advisor noted that graphene samples sent within the Graphene Flagship were tracked and that one always has to be careful in handling materials, in the event that they could have a deleterious effect on one’s health. Here too, the small amount of graphene in use was pointed out: “It will be at least ten to fifteen years before graphene will be involved in the large processes.” One advisor said:

“Graphene is a new area and there are no large amounts bought today, and very little is needed. If all mobile phones’ displays were to be covered with graphene, you would only need five kilos or something.”

Like the scientists, some of the advisors pointed out that graphene is simply carbon: “it’s carbon molecules and we have plenty of carbon, “no (risk involved) – we’re talking about carbon atoms, basically” and “graphene oxidizes at 400 degrees, it burns and becomes carbon dioxide.” One advisor explained:

“[G]raphite has existed all the time – and graphene is basically graphite in many layers, and mixed. People use pencils and children eat the pencils, so you know that it’s probably not supertoxic or anything like that, it’s just carbon.”

Like the scientists, then, these advisors link graphene’s lack of risk to its basis as a common element found throughout nature and currently in common domestic use, such as in pencils.

One advisor brought forward the argument that graphene is not a risk since it is *dissimilar* to the presumed more dangerous carbon-containing material carbon nanotubes:

“You can compare it with the carbon nanotubes. They had some problems a few years back because a few studies appeared which showed that these coal nanotubes weren’t at all beneficial, but rather dangerous, similarly to asbestos […] They are long, and sharp. But graphene doesn’t have that form factor, so to speak.”

### Graphene Is a Risk

Several researchers also expressed that graphene *is* a risk, or at least that risks cannot be excluded, saying for example that “there must be some dangers with graphene” and that “there are risks with graphene therefore we have protection.” Several mention the lack of knowledge as a rationale for graphene being risky, as shown by these quotes: “[…] risk research is at the beginning, you should not eat it [graphene],” “no one knows if graphene is toxic, some say it is other it isn’t,” “there might be health issues but we don’t know yet,” and “it is not clear how toxic graphene can be.” Some mention as rationales that specific conditions must be fulfilled in order for graphene to constitute a risk, in particular related to exposure probability, saying for example “possibly graphene can be dangerous as nano particles, if you breath them in,” “if you insert graphene into your body it might hurt you,” and “it is important to distinguish between flakes that you can breathe in and powder graphene you do not want to inhale.”

Several researchers also say that there are risks related to graphene during production and other product life cycle stages. These are not necessarily related to graphene itself, but possibly to other substances used: “there is a risk with acids that are used in making graphene, but that is easily controlled” and “we use some dangerous chemicals in the process, but they are all approved in the lab.” Two researchers elaborate further:

“The risks vary depending on where the material is in the life cycle. In the manufacturing of graphene in tennis rackets there is a risk of inhaling flake at the beginning. […] [W]hat happens when you throw away electronics? This is looked upon at the moment.”

“Perhaps there are some hazardous chemicals in the manufacturing process. The problem is that the production will be in India and other countries where they are not as thorough. But this is the same for all electronic products. It is during the production and during the recycling there could be risks. We never talk about risk at work.”

Some advisors are also reluctant to conclusively say that graphene is not a risk. The lack of conclusive knowledge about possible risks of graphene is what makes them doubtful, much in the same way as the researchers. They refer to this gap in knowledge in one way or another, while at the same time, maintaining that graphene probably does not constitute a risk:

“We haven’t seen any large risks… But at the same time, one can’t yet say that there aren’t any risks because it’s too large an area, there are too many types of material and too many different systems we have to look at, brain cells, intestinal cells, yes, you know, one has to have done so many studies before one can say that something is bad, or good.”

Another advisor points out:

“If a company has a product, they have to class their materials, they have to account whether it contains ‘hazardous materials’ or such. And then they ask if graphene is a dangerous material, of course they ask. And we have to answer, like, ‘a little, well, maybe, maybe a little, or maybe not’… it’s not poisonous, they haven’t been able to prove that it’s toxic… but it’s just this, there is a lot left to do. That’s what one can say. One has to be pretty honest, and say that it’s not poisonous, but there are many studies left to do.”

Several advisors also adhere to the rationale that graphene constitutes a risk under certain conditions. One advisor points out that although it is composed of carbon atoms, it is not beneficial to inhale. Another notes that difference in health risks are to be expected from different sizes of graphene molecules:

“A large flake that is several micrometers in size, for example, probably has completely different health aspects than a flake which is only, say, 10 or 20 nanometers large. So it’s about size, and how they interact with cells, and other things.”

Later in the interview, this advisor added that research regarding risks should be carried out in parallel with the development of materials and that it was possible that mistakes might be made with graphene, which is why one has to be careful, carry out evaluations, and be vigilant all the time.

One tool that innovation advisors can use to exercise vigilance regarding the possible risks of materials used in new products is the risk assessment carried out routinely when new projects and products are evaluated. In the conversations about risk, four of the six interviewees brought up the importance of these assessments in considering potential problems. One noted:

“It’s always included in any business plan. One mandatory part of the business plan is having the risk analysis… So it’s just a table that has the description of the risk. The probability of the risk, the severity of the risk if it happens, and the actions to prevent the risk happening. So that’s in it, always. But risk as an element is a really important factor of course… when you think about the risk you have to be thinking about the external ones and the internal ones and the ecological ones and the commercial ones, so... because that’s part of the things that are telling you if this is worth doing, but also telling others that you have at least been thinking about these things. So. It’s just not just a list, it’s important.”

This risk assessment is, then, the currently existing means for detecting human and environmental risks implicated by a future widespread use of graphene. Another advisor noted: “We try to include the concept of sustainability in all our ideas, to think about ‘is this environmentally sustainable?’” He continued, however, to say that it is the economic risks that are the most central in the risk assessment. To consider risk and sustainability “is part of the process,” a third advisor said, but noted that if the researcher presenting the proposal did not know of any risks, it was not followed up. This was echoed by his colleague who claimed that they had not thought about the possible risks of graphene, but rather that they had faith in the researchers’ good judgment considering the reviews their research had undergone. As the risk assessment focuses primarily on commercial risks, and the innovators themselves—together with researchers—are the primary providers of the information used in the assessment, it appears the possibility of detecting novel health and environmental risks in the assessments is limited. The advisors are thus constrained to repeating that to date, no dangers have been discovered and adding that more research is needed.

### Additional Ideas

Two ideas expressed by the researchers refer to fundamentally different kinds of risks than the health and environmental risks mentioned above, more related to research and innovation. One said that “the major risk of the graphene is that there is too much money in graphene, other 2D materials are ignored.” The researcher thus suggests that overly high investments in graphene research is hampering important research into other interesting materials and that this is more problematic than any human health- and environmentally related risks. Another researcher said that “the biggest risk is that we will not fulfill the promises around graphene.” This researcher refers to the many promises that graphene brings in terms of innovations (see “Introduction” section) and expresses concerns over what might happen if these high hopes are not realized. It is again implied that this risk is more severe than any health and environmental risks of graphene.

## Discussion

It might seem strange that several researchers and advisors expressed both that graphene *is not* a risk and that it *is* a risk. We see two possible reasons for this seemingly contradictory finding: one rhetorical and one semantic. These are explored below.

As can be seen in Table 1, several graphene researchers and innovation advisors largely adopted a risk/no-risk rhetoric [60] regarding graphene risks. They often expressed that there might be risks, but for various reasons consider these to be nonexistent (e.g., “[graphene] seems to be safe”) or manageable (e.g., “[…]don’t insert it into the body”). Sometimes, the researchers and advisors interviewed instead stated that graphene is not a risk, then admitted that there might be some (types of) risk, possibly given certain conditions (e.g., “[…]there is risk with acids [not graphene itself]” and “[…]graphene can be dangerous […] if you breath them in”). Corvellec and Boholm [60] concluded that the environmental impact assessments conducted for wind power farms should not be seen as objective or neutral reports but rather as contributions that serve a particular interest; specifically, in that case, the reality that wind farms can largely be disconnected from any risks. We can understand the graphene researchers and innovation advisors seemingly contradictory responses in a similar way: although they seem to agree that there is insufficient knowledge about graphene risks, they have an interest in contributing to the idea that graphene is not a risk, therefore dissociating it from risk or at least limiting the association to certain situations. Moreover, from the viewpoint of the researchers, graphene is something familiar, a view that results from everyday interaction with it in their work. As such, their relationship to graphene is likely to be an affectionate and personalized one, including a feeling of control, rather than regarding it as threatening or risky [41]. The additional primary ideas, where some respondents worry more about the lack of funding for other two-dimensional materials and the possible lack of fulfillment of graphene’s promise than they do about graphene’s impact on human health and the environment, strengthen this interpretation.

**Table 1:** Risk/no-risk statements by the graphene researchers and innovation advisors. Risk statements that should perhaps rather be classified as hazard statements according to the risk-hazard dichotomy are marked with an H within brackets

Risk	No-risk
Graphene researchers	
“There must be some dangers with graphene…”	“…but it is a safe material… It is not toxic.”
“There might be health issues but we don’t know yet.”	“But it seems to be safe.”
“If you insert graphene into your body it might hurt you.” (H)	“But you do not insert it into the body.”
“It is important to distinguish between flakes that you can breathe in and powder graphene you do not want to inhale. What happens at the edges of graphene is important because other atoms can interact and disrupt cells.” (H)	“So far, the [Graphene] Flagship could not see any acute toxic effects… We see nothing alarming.”
“There are also health risks which limit its usability…”	“…but I am not aware of any such risks yet.”
“There is a risk with acids that are used in making graphene…”	“…but that is easily controlled.”
“We use some dangerous chemicals in the process…” (H)	“…but they are all approved in the lab.”
Graphene innovation advisors	
“We have to study it… we do extensive studies.”	“We have not found any risk so far.”
“When they [companies] ask if it’s dangerous we have to say ‘a little, well, maybe, maybe a little, or maybe not’.”	“It’s not poisonous, there’s no danger that it’s a toxin.”
“It’s too large a research area to say that there are not any risks… we have to do so many more studies.”	“So far one has not seen any large risks, on the contrary, the toxicologists have not found anything.”
“It’s not good if you breathe it in…” (H)	“…but it’s just carbon atoms.”
“Research has to be conducted in parallel with the development of new materials and ways of using them.”	“One cannot think of everything, but the risk should be quite small unless I am wrong.”

Considering the semantic explanation, we can first note the conceptual distinction between risk and hazard that is often made in the risk research literature. Hazard then refers to potential loss, while risk refers to exposure to a hazard [61, 62]. “Risk is the actual exposure of something of human value to a hazard and is often measured as the product of probability and loss” [63]. This reasoning reflects Paracelsus’ basic principle of toxicology, namely, that the dose makes the poison, implying that potentially harmful substances (i.e., hazards) kept safe are not risks. Accordingly, the actual harm of a substance cannot be determined independently of considering the exposure of the organism at stake to the substance. Even severe toxins (e.g., mercury and arsenic) only constitute actual risks given that the dose is large enough. Conversely, any substance can have harmful effects on the human body and the environment given large enough doses.

However useful and clear this theoretical distinction between risk and hazard might be, the word risk has a less narrow and precise meaning in common usage [64]. In fact, the word risk is used both in the sense of hazard (i.e., as a potential loss) and in the sense of risk (i.e., as exposure to a hazard), as these terms are defined in the distinction. For example, the Oxford English Dictionary [65] reflects this ambiguity of the noun risk (rather than disambiguating it), when identifying “(Exposure to) the possibility of loss, injury, or other adverse or unwelcome circumstance” as one of the senses of the word. This polysemy of the word risk partly explains how claims can be made that graphene is a risk by the same person who also claims that graphene is not a risk. Graphene can be identified as a risk in terms of a possible source to harm (i.e., a hazard) due to substantial uncertainty and given certain conditions (e.g., bodily exposure), but not necessarily as a (proper) risk in terms of an exposure to harm (e.g., due to safety procedures and/or limited production). A closer look at the risk statements in Table 1 suggests that some of them should perhaps rather be called hazard statements following the risk-hazard dichotomy described above. As shown in Table 1, at least four of the statements refer to inherent hazard-type harmful properties of graphene, such as toxicity, rather than actual risk. This suggests that some of the statements are hazard/no-risk statements rather than risk/no-risk statements, indicating that there may be some truth to the semantic explanation.

The semantic explanation does not necessarily point to the respondents being irresponsible from an RRI point of view, but rather to the semantic difficulty (i.e., polysemy) of the risk concept in common language [66]. However, the rhetorical explanation points at the possibility that graphene researchers and innovation advisors may be downplaying graphene risks, despite existing risk-related research [23, 28, 29, 31]. Given this explanation, some of the current ideas about graphene risks held by graphene researchers and innovation advisors, in particular the view of graphene as “just carbon”, seem to constitute challenges for RRI in the graphene field. However, we can at the same time observe some kind of ambivalent responsibility among the respondents. In light of the current lack of knowledge about nanomaterial risks in general [24–26] and graphene risks in particular [11, 23], this ambivalence is not unreasonable. Considering the increased importance of RRI in technological development and innovation projects [21], there are strong expectations that central actors in technological development and innovation should acknowledge the possibility of risks. From this perspective, the respondents are *responsible* if they associate graphene with risk, which they partly do. However, the respondents are at the same time *irresponsible* to associate graphene with risk, since they clearly engage in the then-risky activity of graphene development and innovation on an everyday basis. Demands for responsibility thus also motivate the respondents to dissociate graphene from risk and to downplay risks, which they also do. Given this dilemma-like situation, it is perhaps no wonder that the respondents express a mixed understanding of graphene as a possible risk. Existing research policy on RRI from the European Commission [67] provides little guidance on this concrete and individual-level matter, but rather focuses on six quite vague dimensions (multi-actor and public engagement, gender equity, science education, open access, ethics, and governance models). Existing scientific frameworks for RRI typically focus on (more over-arching) research policies and projects, rather than on individual persons, when evaluating responsibility [17, 68].

We therefore recommend further research into how RRI should be approached in practice, in particular what actors such as researchers and innovation advisors should do (or think) to qualify as responsible. Potentially interesting research questions include:

- Which role should scientists and innovation advisors have in the early anticipation and avoidance of risks?
- Do scientists and innovation advisors experience a ‘damned if you do, damned if you don’t’ dilemma when it comes to acknowledging technological risks?
- Can individual-level criteria for RRI be formalized?

Our recommendation is in line with Frankel [69], who writes that there is a lack of research about what the social responsibilities of researchers are and how they should be operationalized. An interesting study in the spirit of our recommendation was conducted by Glerup et al. [70], who noted that although the researchers they interviewed did not consider responsibility (as understood in an RRI context) to be of importance to them, they still exercised a number of responsible practices, such as producing excellent and robust science, taking care of employees and conducting publicly legitimate research.

## Conclusion

We have found that the main idea held by graphene researchers and innovation advisors is that graphene is not a risk. Rationales provided for this are that graphene is not toxic, that exposure is low, that small amounts are expected to be produced and used, that graphene can be made safe, that graphene is similar to harmless materials (e.g., being “just carbon”), and that graphene is different from hazardous materials such as carbon nanotubes. A less frequent but still common idea is that graphene is a risk. This is motivated by the current lack of risk-related information for graphene, as well as that it can be risky under certain conditions and in different stages during its product life cycle (e.g., production and waste treatment). We suggest that these two seemingly contradictory ideas can be explained through (1) the semantic ambiguity of the word risk, which can denote both actual and potential losses, or (2) a risk/no-risk rhetoric, during which risks are first rhetorically mentioned only to be disregarded as manageable or negligible later. We also note that some researchers mentioned as main risks that the focus on graphene could hamper research and development of other interesting two-dimensional materials, and that the high promises of graphene may not become fulfilled. Given the second explanation, we suggest that some of these ideas held by the graphene researchers and innovation advisors constitute challenges for RRI in the graphene field and for graphene technology. At the same time, we acknowledge that the uncertainty of graphene risks and the RRI discourse put the respondents in a dilemma-like situation. If they deny that graphene is a risk, they become irresponsible because of their lack of risk awareness. If they affirm that graphene is a risk, they also become irresponsible because of the engagement in graphene development that their work constitutes. More research regarding what researchers and innovation advisors should do in practice in order to qualify as responsible is therefore recommended.
